# Advances in the Histone Acetylation Modification in the Oral Squamous Cell Carcinoma

**DOI:** 10.1155/2023/4616682

**Published:** 2023-02-09

**Authors:** Ying Lu, Jinjin Yang, Junwen Zhu, Yao Shu, Xuan Zou, Qiao Ruan, Shuyuan Luo, Yong Wang, Jun Wen

**Affiliations:** ^1^School of Stomatology, Southern Medical University, Guangzhou 510515, China; ^2^Department of Stomatology, The Fifth Medical Center of PLA General Hospital, Beijing 100071, China; ^3^Harbin Medical University Cancer Hospital, Harbin, Helongjiang 150081, China; ^4^Stomatological Hospital, Southern Medical University, Guangzhou 510280, China

## Abstract

Oral squamous cell carcinoma (OSCC) is one of the common malignant tumors in the head and neck, characterized by high malignancy, rapid growth and metastasis, high invasive ability, and high mortality. In recent years, surgery combined with chemotherapy or radiotherapy remains the preferred clinical treatment for OSCC, despite considerable advances in diagnostic and therapeutic techniques. Hence, new targeted therapy is urgently needed. Histone modification affects the function of massive cells through histone acetyltransferase and histone deacetylase. Accompanied by the progress of some diseases, especially tumors, these proteins often show abnormal functions, and by reversing these abnormalities with drugs or gene therapy, the cancer phenotype can even be restored to normal. As a result, they are potential drug targets. This article reviewed the role of the histone dynamic process of acetylation modifications and their associated active modifying enzymes in the pathogenesis and progress of OSCC. Moreover, we explored the value of histone acetylation modification as a potential therapeutic target and the new progress of related drugs in clinical treatment.

## 1. Introduction

Oral cancer is one of the most common malignancies occurring in the maxillofacial region, with a mortality rate close to 50% and an overall 5-year survival rate of about 60%. 90% of them are squamous cell carcinoma. Oral squamous cell carcinoma (OSCC) is the sixth most common malignant tumor in the world. It occurs mostly in adults aged 40‒60 years in China, with more males than females. Alcohol, tobacco, and HPV infection are the most important risk factors for OSCC [1]. In recent years, the conventional therapies for OSCC have evolved into various modalities, including surgery, chemotherapy, radiotherapy, and combination therapy based on the diagnostic stage [2], but none of them has brought significant outcomes to the prognosis of OSCC. This is mainly due to the special anatomical structure of the oral cavity that provides great ease for metastasis. Most of the administered patients with progression to the middle and late stage have already missed the best treatment opportunities, with serious prognostic implications. Hence, the early diagnosis, treatment, and inhibition of metastasis are critical to control this disease. Moreover, it has great clinical significance to investigate the molecular biological mechanisms of occurrence, development, and metastasis of the disease and to discover new therapeutic targets for the intervention in those critical molecules and processes to achieve early diagnosis and treatment and suppress metastasis.

**Figure 1:**
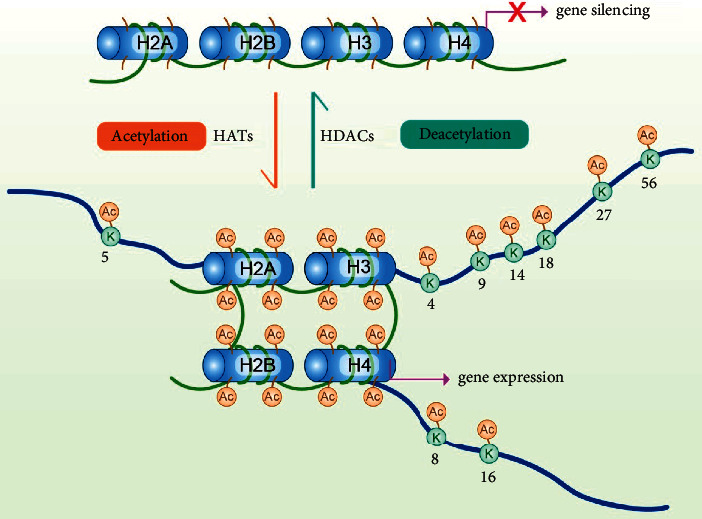
The dynamic process of reversible acetylation. Commonly functioning acetylation sites in oral squamous carcinoma.

The progression of OSCC is mainly caused by the genetic or epigenetic alterations in proto-oncogenes, antioncogenes, and dysregulation of molecular networks. Histone modification is one of the most critical epigenetic regulations for gene expression by posttranslational modification of the histones [3, 4]. Posttranslational modifications of proteins are essential for organisms, while the metabolism and metabolites can control the activity of growth-related signaling pathways to regulate cell growth through covalent modifications of proteins [5]. The histone acetyltransferases (HATs) and the histone deacetylases (HDACs) are responsible for controlling the histone acetylation level and chromatin accessibility [3, 6]. Acetylation of the *ɛ*-amino group on the histone lysine residues plays a key role in cell growth activity and gene transcription. Epigenetic and posttranslational modifications have emerged as new targets for anticancer therapy. This review focused on the relationship between the acetylation/deacetylation of histones and the OSCC and the potential therapeutic targets.  Figure 1 shows the pattern of acetylation function and lists the reported sites in OSCC.

## 2. Histone Modification

The concept that epigenetic abnormality is a possible sign of cancer has now been verified over the past decades. Several studies have suggested that the multimutations and the copy number alterations in the epigenetic modifiers (including acetyltransferase, deacetylase, methyltransferase, demethylase, and kinase) might jointly promote the progression of OSCC [7]. The nucleosome made up of approximately 147 bp of DNA is the basic unit of chromatin. It envelops a 2-copy histone octamer containing 4 kinds of histones (i.e., H2A, H2B, H3, and H4). Chromatin-modifying enzymes dynamically perform the posttranslational modifications of histones and DNA through tightly regulated mechanism [4, 8]. Posttranslational modification of histone is also an important mechanism regulating the structure and function of chromatin, which acts as a critical role in the emergence and progression of cancer through regulating the genetic transcription, chromatin remodeling, and nuclear structure [9]. Various posttranslational modifications exist in the eukaryotic animal cells, which usually include phosphorylation, ubiquitination, glycosylation, methylation, and acetylation. The histone tail projection of the nucleosome octamer experiences several posttranslational modifications including the added chemical groups like acetyl, phosphate, and methyl. Less common modifications include ubiquitination, sulfonation, and ribosylation, occurring on the residues of lysine, arginine, and serine on histones [10].

### 2.1. Acetylation

Various posttranslational modifications can regulate the function of histones, including the reversible acetylation of lysine terminal 1 group on histones. Acetylation is a universal protein modification that modulates a variety of cellular events including the cell cycle, cell metabolism, gene transcription, signal transduction, and RNA splicing [11]. Histone acetylation can disrupt or remove the nucleosomes from the transcription region and loosen the chromatin to facilitate the entry of proteins to the DNA to be replicated or transcribed. Studies of proteomics stated that acetylation can occur in every corner of the cell on thousands of proteins [12]. Histone acetylation is a widely studied posttranslational modification that can be reversed through the transfer of acetyl groups to lysine residues by HATs and its further removal by HDACs.

Histone acetyltransferase can attach the acetyl groups to the lysine residues in both histones and nonhistones, which can be divided into 2 groups according to their intracellular location and substrate specificity. The first group, named HAT, is located only in the nucleosomes and that has the effect on chromatin remodeling by modifying the chromatin-related histones. The other group, named B HAT, is located both in nucleosomes and cytoplasm and that can acetylate free soluble histones [13]. The common HATs are mainly composed of three families: the GCN5/PCAF family, the CBP/p300 subfamily, and the MYST family (MOZ, MOF, TIP60, and HBO1) [14] (see Table 1). The largest subfamily of histone acetyltransferase (MYST family) contains a high conserved MYST domain, and this structure consists of a zinc finger and an acetyl-CoA binding motif. The histone acetyltransferase has some extra structures including the plant homologous structural domain-linked zinc fingers (MORF and MOZ) and chromophores (TIP60 and MOF) [15], which are parts of the protein-complexes involved in the pro- and anticancer activities [16]. Another group of N-acetyltransferase (GNAT) family related to GCN5 is represented by the p300/CREB binding protein (CBP), binding factor (PCAF), HAT1, and GCN5, which contains bromo polysaccharides and adds an acetylated group of lysine to histone H2B, H3, and H4 [17, 18]. The CBP/p300 subfamily contains many small domains that interact with many other proteins containing disordered domains, including p53 and NF-*κ*B [19].

HATs catalyze the transfer of acetyl groups to the target lysine (Lys/K) residues on the histone tail, thereby neutralizing the positive lysine charge, loosening condensed chromatin, promoting activation of gene transcription, and exposing binding sites for “binding motif proteins” that recognize the modifications on the histone [20]. Apart from the histone charge modification, histone acetylation can regulate the intracellular pH. Interestingly, many tumor cells show low histone acetylation levels and the acidic internal environment. Besides, poor prognosis in cancer patients has also been associated with low intracellular pH [21]. Different HATs can simultaneously act as tumor suppressors and oncogenes, which mean the balance of acetylation is critical to the stability of cells [22]. For example, as a kind of HAT, Tip60 can regulate ataxia-telangiectasia mutated (ATM) and DNA damage response pathways to participate in tumorigenesis, and it can also activate the transcription of p53 and Myc. ATM is a key regulator of DNA double strand break repair. Upregulated Tip60 can activate ATM through acetylation to promote DNA damage repair and inhibit tumor cell growth [23]. The reduction of Tip60 expression leads to genomic instability and apoptotic signaling cascade impairment, promoting the onset of malignant transformation of tumors [24] (see Tables1 and 2).

### 2.2. Deacetylation

Contrary to the HATs, the HDACs remove acetyl groups from the high acetylated histones and repress the gene transcription. There are 4 HDACs in mammals, including the Zn^2+^-dependent class I (i.e., Rpd3-like enzyme) consisting of HDAC1, HDAC2, HDAC3, and HDAC8; the class II (i.e., Hda1-like enzyme) consisting of a subclass of IIa (HDAC4, HDAC5, HDAC7, and HDAC9 belong to class IIa) and IIb (HDAC6 and HDAC10 belong to class IIb); the class III (i.e., Sir-like enzyme) consisting of 7 SIRTs referred as the NAD-dependent deacetylase; the class IV of only HDAC11 with homologous sequences of class I and II [24](see Table 2).

HDAC regulates tumorigenesis through mechanisms like activating oncogene signaling pathways and downregulating tumor suppressor genes. HDACs have low substrate specialty with each kind of them deacetylating multiple sites on histone. Although mutation of HDACs is rare, overexpression of HDACs is still common in cancer [10, 25]. HDACs interfere with the transcription of oncogenes and antioncogenes by removing the acetyl groups from histones and reversing the acetylation of chromatins. Furthermore, HDACs can catalyze the deacetylation of many nonhistone proteins, which control a series of biological processes including the development and progression of cancer [26]. These processes are involved in the cell cycle, apoptosis, DNA damage response, metastasis, angiogenesis, autophagy, and other honeycomb shaping process [27].

### 2.3. Histone Acetylation and Oral Cancer

Accumulating scientific evidence have presented that epigenetic alterations, including chromatin remodeling, noncoding RNAs, DNA methylation, and histone covalent modifications, are generally involved in oral carcinogenesis and treatment tolerance. Epigenetic modifications contribute to the formation of cell plasticity and cancer stem cells (CSCs) during tumor progression [3]. Especially the dysregulation of histone acetylation leads to the disorder of the activity of different genes, resulting in malignant transformation-related events. Due to the reversibility and low-abundance of acetylation, it is a challenge to identify a large number of acetylation sites. Few literature have indicated changes in histone acetylation on specific lysine residues in head and neck squamous cell carcinoma (HNSCC) [28]. The reported main acetylation sites are listed in Table 3.

Most theories confirmed that in HNSCC, low level histone acetylation makes the nucleus smaller, which reduces the DNA damage repair proteins flowing into the nucleus and enhances the resistance to intercalation agents [34], with the most studied being H3K9ac. Some studies have demonstrated that the deletion of histone H3K9ac indicates the occurrence of chemoresistance and is associated with the NF-*κ*B signaling and the accumulation of CSC [35]. The decrease of H3K9ac is related to the activation of epithelial-mesenchymal transition (EMT) and the increased cell proliferation during the process of oral carcinogenesis, indicating the H3K9ac is involved in the progression of HNSCC and is coexpressed with the mesenchymal vimentin prior to the invasion of HNSCC [30]. In oral cancer, the low level of H3K9 acetylation indicates a bad prognosis. The expression level of H3K9Ac is expressed at lower levels in OSCC than in oral leukoplakia, an oral precancerous lesion. In survival analysis, low expression of H3K9Ac was associated with a poorer prognosis in OSCC [31]. Interestingly, in contrast, some studies have found the hyperacetylation of histone H3 (mainly H3K14 and H3K9) in samples from patients with OSCC as well as the hyperacetylation of H2AK5 and H3K56 and hypoacetylation of H4K8 and H4K16 [29]. Several reports have reported the acetylation of special lysine residues in oral cancer. For example, both low and high levels of H3K4ac have been reported to be correlated with the progression and the poor prognosis of OSCC. The hypoacetylation of H3K4 is associated with the advanced OSCC tumor staging, primary tumor (*T*), regional lymph nodes (*N*), and perineural infiltration (PNI), while the H3K18ac is positively correlated with tumor stage [28]. Upregulation of H3K27ac was found to be associated with the activation of the placenta-specific protein 2 (PLAC2) gene in OSCC. PLAC2 promoted the progression of OSCC by modulating the Wnt/*β*-catenin signaling pathway. Compared with normal oral epithelial keratinocytes, PLAC2 is more abundant in oral squamous cell carcinoma cells (CAL-27 and SCC-9) [33]. Moreover, the non-long-strand coding RNA lncRNA MX1-215 can directly interact with the H3K27 acetylase GCN5, interrupting the combination of GCN5 and H3K27, interfering the transcription of PD-L1 and LGALS9 mediated by the acetylation of H3K27, and decreasing the acetylation level and expression level of PD-L1 and galectin-9 on the tumor cells [32]. It is also found that the hMOF (males absent on the first) can participate in the activation of transcription through acetylation of H4K16 and regulate the growth of cancer cells by the multicomb histone enhancer of zeste homolog 2 in OSCC. The upregulation of hMOF indicates poorer overall survival and disease-free survival [36].

### 2.4. Histone Deacetylation and Oral Cancer

Changes in the expression of multiple HDACs have been reported to be closely related to the regulation of cell cycle-related genes, cell invasion, apoptosis, angiogenesis, differentiation, and migration in a variety of cancers [37, 38]. For example, several studies have shown that the HDAC2 is overexpressed in OSCC, leading to increased stability of HIF-1*α* and the increased invasion and migration of HNSCC. The high abundance of HDAC2 is associated with the *T* and *N* states in the late stage [39]. Sakuma et al. [40] also found that HDAC6 is overexpressed in late HNSCC, indicating the activity of HDAC6 may associate with the tumor invasiveness of oral cancer. Besides, some researchers also found that HDAC6 catalyzes the deacetylation of *α*-tubulin and increases cell mobility and tumor metastasis [41]. The HDAC7 is also overexpressed in head and neck tumor, whose accumulation can activate proto oncogene c-MYC and promote cell proliferation [42]. The accumulation of HDAC8 induces the proliferation of cancer cells by inhibiting the activation and autophagy of Caspase in OSCC. In contrast, silencing of HDAC8 significantly inhibited OSCC cell proliferation, invasion, and metastasis [42]. HDAC9 has been reported to have oncogenic effects in OSCC by targeting proapoptotic genes to promote tumor growth. The low expressed HDAC9 inhibited the proliferation of SCC116 cells, increased apoptosis, and induced the *G*0/*G*1 arrest. The overexpressed HDAC9 positively correlates with the OS and promotes OSCC by targeting the transcription factors of MEF2D and NR4A1/Nur77 (proapoptotic MEF2 targets) [43].

The SIRT family belongs to the NAD^+^-dependent histone deacetylase class III and is involved in the cell cycle, transcriptional regulation, and metabolism [44]. Contrary to the HDAC, the SIRT acts as a tumor suppressor in cancer, preventing the occurrence of DNA damage and oxidative stress [45]. The SIRT1, SIRT2, SIRT3, SIRT5, and SIRT7 are reduced in advanced HNSCC and have the potential to be used as prognostic markers [46]. While SIRT6 accumulates in peripheral blood of HNSCC patients [47], SIRT1 is the mammalian homolog of the chromatin silencing factor Sir2 in *S*. *cerevisiae*, which is expressed at elevated levels and catalytic activity in OSCC cells [48]. The latest study has presented that the SIRT7 inhibits the EMT in the metastasis of OSCC by promoting the deacetylation of SMAD4, therefore, reducing the proliferation and invasion of OSCC cells in vitro [49]. In OSCC, the posttranscriptional regulation of SIRT3 is induced by miR-31, which targets the SIRT3 to destroy the mitochondrial structure and increase the oxidative stress response in oral cancer. The downregulation of SIRT3 reduces the migration and invasion of cancer cells enhanced by miR-31 [50]. The reported main histone deacetylase are listed in Table 4.

## 3. The Role of Histone Deacetylase Inhibitors in the Treatment of Oral Squamous Carcinoma

Owing to the reversibility of acetylation modifications, it is clear that the changes observed in the development of cancer have become attractive targets for therapy with the thriving research on the histone deacetylase inhibitor (HDACi)-related drugs. Although the HDACi has been applied to the treatment of hematologic malignancies successfully, its clinical efficacy as an independent drug in the treatment of solid tumors remains limited. The HDACi is able to achieve the best clinical efficacy only by combining with other treatments, including radiotherapy and chemotherapy, for synergistic or additive effects [51]. Current research data suggested that the HDACi has the best effect on HNSCC when administered together with other therapeutic drugs [52, 53].

The HDACi counteracts the abnormal acetylation state of proteins in cancer cells and reactivates the expression of tumor suppressors, thus inducing cell cycle arrest, apoptosis, differentiation, and inhibiting angiogenesis and metastasis [8]. Besides, the tumor cells are more sensitive to the apoptosis induced by HDACi than normal cells [54]. Based on their chemical structures, HDACi is classified into four classes, including hydroxamic acid, cyclic peptides, short-chain fatty acids, and benzamide. Most of them have already been developed as anticancer drugs with different specificity, efficiency, and pharmacokinetic and toxicological properties [8, 51–53, 55, 56]. Studies on the anticancer effects of HDAC in oral squamous cell carcinoma are presented in Table 5.

### 3.1. The Class of Hydroxamic Acid

The hydroxamic acid class of HDACi has been extensively studied in recent years, which mainly consists of rings, aliphatic chains, and hydroxamic acids for the surface recognition region, linkage region, and metal binding region (zinc combining region), respectively. It mainly contains SAHA (suberoylanilide hydroxamic acid), trichostatin A (TSA), and panobinostat, belinostat.

SAHA is a nonselective HDACi and the first FDA-approved HDACi that can be used alone or in combination as clinical treatment since October 2006 [77]. A research report found that SAHA can enhance the acetylation of H2A and H3 and inhibit the anticancer activity in vitro and xenotransplantation models by inducing cell activity reduction, caspase dependent apoptosis, and tumor growth inhibition [57]. In addition, powerful antiproliferative and synergistic effects of SAHA and gefitinib were noted. The SAHA enhances the antitumor function of gefitinib by upregulating the E-cadherin and ErbB3, downregulating the vimentin, EGFR, and ErbB2, and the restoration of mesenchyme to an epithelial phenotype [58]. Studies also reflected that the SAHA can enhance the cellular chemosensitivity to cisplatin, thereby mediating apoptosis in oral squamous carcinoma cells [78].

It has been verified that TSA is a natural inhibitor of HDAC class I and II. The changes in chromatin acetylation of HNSCC unexpectedly triggered a decrease in CSC. The hyperacetylation of chromatin in HNSCC induced by TSA can interrupt the generation of CSC and destroy the ability of stem cells to maintain tumor globules. It is also demonstrated that the TSA can decrease the enzyme activity of ALDH (a recognized marker of CSCs) [30]. The SAHA and TSA also reduce the CSC markers of CD44 and ABCG2, the expression of genes related to cell stemness, and the EMT phenotype in the oral cancer [79]. The combined application of TSA and PS-341 (bortezomib), a proteasome inhibitor, was also studied in HNSCC. Although TSA alone did not induce apoptosis, it can activate cystathionine to significantly enhance the degree of apoptosis induced by PS-341 [59]. The combination therapy of TSA and all-trans retinoic acid (ATRA) synergistically inhibited the growth of tumor cells and strongly induced transcriptional and activation of target genes, thereby restoring the tumor sensitivity of HNSCC cell lines to retinoic acid [60]. HDACi reportedly appears to be valuable in the treatment of radiosensitized tumors in solid tumors, including HNSCC. TSA, SAHA, M344 (SAHA analogue), and desmethyl peptide (FR90228) regulate the cellular response to ionizing radiation and promote apoptosis and cell cycle arrest in HNSCC [80]. Recent treatments have shown that the TSA combined with 5-Aza-dC or RG108 can significantly reduce the vitality of HSC-2 cells and Ca922 cells. The TSA combined with DZNep also reduces the vitality of Ca922 cells, and the combination of TSA and the combination of TSA with other epigenetic inhibitors is also therapeutic for OSCC [81].

Panobinostat, an FDA-approved hydroxamic acid analogue (LBH589) for the treatment of refractory/recurrent multiple myeloma, induces hyperacetylation of histones *H*3 and *H*4 [82]. The LBH589 can inhibit cell growth and induce the *G*1 arrest and the apoptosis of OSCC by increasing the inhibition of transcription factor specificity protein 1 [61]. In vitro studies performed by flow cytometry on laryngopharyngeal (FaDu) and oral (CAL-27, SCC-15, UM-SCC-1, and UM-SCC-47) OSCC cell lines showed that treatment of these cells with LBH589 upregulated p21 and induced G2/M phase block and cell death [62].

### 3.2. The Class of Cyclic Peptide

The cyclic peptide class of HDACi can be divided into 2 categories according to the functional groups: one category contains (2S, 9S)-2-amino-9, 10-epoxy-8-oxodecanoic acid (Aoe), and epoxy ketone, while the other category does not contain the Aoe structure, which is mainly represented by apicidin, FK228, and trapoxin.

Apicidin is a cyclic tetrapeptide isolated from *Fusarium*, whose decanoic acid side chain, macrocyclic structure, and tryptophan side chain competitively bind HDAC. It has a significant antitumor function involving the antiproliferative activity and differentiation inducing activity toward the cancer cells [63]. Moreover, the OSCC cell lines treated with apicidin presented elevated levels of LC3-II, *G*2/*M* arrest, apoptosis, and autophagy as assessed by the MTT, DAPI staining, and flow cytometry [64].

Romidepsin (FK228) is a natural HDACi separated from *Chromobacterium violaceum*, which can induce the inhibition of HDAC with the characteristics of p21 (Waf1/Cip1) and reduce the staining of Ki67. Despite the limitations of single romidepsin treatment in the treatment of HNSCC, it still can effectively inhibit the tumor-related HDAC [65]. As an HDAC inhibitor, the FK228 induces the telomerase reverse transcriptase (hTERT) gene through a complex mechanism that involves the inhibition of histone deacetylation and other transcription factors except for the c-myc [66].

Trapoxin, a fungal product, was found to induce morphological reversion from transformed to normal in sis-transformed NIH3T3 fibroblasts [83]. Low concentration of trapoxin irreversibly inhibited the deacetylation of acetylated histone molecules through the covalent binding of epoxides and histone deacetylase. The process caused accumulation of highly acetylated core histone in a variety of mammalian cell lines [84]. Other findings have found that the product of binding trapoxin to histone deacetylase HDAC8 helps inhibit tumor cell proliferation as a potential anticancer compound [67].

### 3.3. The Class of Short-Chain Fatty Acid

The short-chain fatty acid class of HDACi has a relatively simple structure including a carboxyl group that binds the metal ions. It mainly contains sodium butyrate, valproic acid, and phenylbutyric acid.

Valproic acid (VPA), which is often widely used as a broad-spectrum antiepileptic drug and mood stabilizer in clinical practice, has been reported to be a class I and II HDAC inhibitor that promotes hyperacetylation of the *N*-terminal chains of histones *H*3 and *H*4 and nonhistones, thereby altering chromatin structure and preventing restricted expression of oncogenes. Moreover, several researchers have reported that the enhanced function of HDAC can stabilize the DNMT, while the VPA degrades the DNMT1 by multiple biochemical mechanisms including acetylation [68]. Because of its HDAC inhibitory activity, VPA has been a safe treatment for epilepsy for many years, and therefore, VPA is considered a good candidate for antitumor therapy in patients with metastatic or recurrent HNCs. In a clinical study, Gan et al. [52] have found that VPA inhibits the growth of HNSCC cell lines as a monotherapy or in combination with other anticancer drugs at physiological doses. The VPA therapy can increase the *G*1 arrest, apoptosis, and the expression of small ubiquitin-related modifiers. VPA treatment resulted in reduced tumor volume and increased apoptosis in xenograft models [69].

As an ammonia scavenger in the treatment of urea cycle diseases, phenylbutyric acid (PBA) leads to cell apoptosis, differentiation, and cell cycle arrest. Radiation-exposed patients receiving PBA have relatively low levels of mucosal oxidative stress and TNF-*α*, accompanied by mild oral mucositis, which promotes DNA repair and survival [71]. Sodium phenylbutyrate has a proapoptotic effect on tumor cells, inhibiting transforming growth factor-*β*-related epithelial-mesenchymal transition, with a decrease in the mesenchymal marker N-calmodulin and an increase in the epithelial marker E-calmodulin [70]. The PBA-derived HDACs (S)-HDAC42 present a higher activity of antiproliferation than SAHA, which can induce cell apoptosis by elevating the p21and p27, reducing the levels of CDK6 in the *G*1 phase, the cyclin D1 and the phosphorylation of Akt. Accordingly, it showed high potency in inhibiting the growth of OSCC in a Ca922 xenograft nude mouse model [85].

The short-chain fatty acid derivative sodium butyrate (NaBu) is another class I and IIa HDAC inhibitor belonging to aliphatic fatty acids. It is presented by the flow cytometry and immunocytochemical analysis that the NaBu significantly inhibits the proliferation of Tca8113 in a time and dose-dependent manner, and the NaBu therapy can inhibit the in vitro growth of OSCC cell lines and induce the cell cycle arrest, which may be related to the increased expression of p27 [72].

### 3.4. The Class of Benzamide

The benzamide class of HDACi has not been extensively studied as the first three HDAC inhibitors, which mainly contain the MS-275 (entinostat) and MGCD0103 (mocetinostat).

Entinostat is a bioavailable class I HDACi with a long half-life. In vitro and in vivo studies suggested that entinostat alone or in combination may be a promising agent for the treatment of OSCC due to its antiproliferative and proapoptotic effects. Administration of entinostat could reduce the proliferation of OSCC cells, leading to the *G*0/*G*1 arrest and massive apoptosis of tumor cells. Meanwhile, an increase in reactive oxygen species and a significant reduction in CSCs was observed as well. Entinostat also caused an increase in acetylated histone *H*3 or *H*4 and changes in the expression of cell cycle-related proteins (e.g., p21) [73]. The synergistic effect of HDACi and cisplatin enhanced the induction of cytotoxicity and apoptosis compared with using cisplatin alone, while the MS-275 also played the same role in the cytotoxicity of cisplatin during the treatment of OSCC. [74]. Combination treatment also activated the miR-138 and miR-107, leading to interrupting the following migration and invasion of tumor cells [75].

Preprocessing of the OSCC cell lines with the MGCD0103 and 5-aza20-deoxycytidine prior to radiotherapy resulted in better radiosensitization compared to the preprocessing by 5-Aza-dC alone [76].

## 4. Summary and Outlook

Histones have many common epigenetic modifications, and acetylation is one of the most common, which regulates DNA transcription and interferes with gene expression. The deacetylation is opposite of acetylation. The balance of those two reactions is necessary to ensure the integrity of chromatin. Therefore, histone acetylation in cancer cells can play a double role in cancer progression, which may be involved in suppressing the silencing of oncogenes and enhancing the expression of oncogenes. Evidence accumulated from a large amount of experimental data suggests that overexpression of HDAC may be closely associated with the development and progression of OSCC, and HDAC can be considered as a potential anticancer agent for OSCC. Further study on histone acetylation modification may be helpful to discover new therapies for oral cancer.

## Figures and Tables

**Table 1:** The major HAT families.

Family	Dominant members	Characteristics
GANAT	HAT1	Contains bromo polysaccharides and conducts the acetylation of lysine on histone H2B, *H*3, and *H*4
	GCN5	
	PCAF	

MYST	TIP60	Contains a high conserved MYST domain, which consists of a zinc finger and an acetyl coenzyme, a binding motif
	MOF	
	MOZ	
	MORF	
	HBO1	

P300/CPB	P300/CPB	Contains many small domains that interact with many other proteins containing disordered transactivation domains

**Table 2:** The major HDAC families.

Family	Dominant members	Characteristics
I	HDAC1, 2, 3, 8	Widely expressed in human cell lines and nuclear tissues
II	IIa: HDAC4, 5, 7, 9	Tissue specific expression, shuttling between nucleus and cytoplasm
	IIb: HDAC6, 10	
III	Sirt1, 2, 3, 4, 5, 6, 7	NAD^+^ dependent, with a very unique catalytic mechanism for deacetylation
IV	HDAC11	Deacetylate different histone sites, resulting in reduced substrate specificity

**Table 3:** Histone acetylation sites and expression in OSCC.

Sites	Expression	Acetylase	Function	References
H2AK5	Hyperacetylation	Tip60	Hyperacetylation is associated with cancer promotion	[29]
H3K4	Hypoacetylation	P300/Tip60	Low acetylation level is positively correlated with malignancy	[28]
H3K9	Hypoacetylation	P300	CSC accumulation leads to hypoacetylation and promotes tumor; its absence is a hallmark of chemoresistance	[30, 31]
	Hyperacetylation		High acetylation expression detected in oral cancer	[29]
H3K14	Hyperacetylation	MOZ/MORF	P300-mediated hyperacetylation promotes tumor growth	[29]
H3K18	Hyperacetylation	P300	High acetylation levels are associated with poor prognosis	[28]
H3K27	Hyperacetylation	GCN5	Hyperacetylation enhances PD-L1 expression to promote tumor infiltration, metastasis, and recurrence	[32]
	Hyperacetylation		Hyperacetylation activates PLAC2 to promote tumor proliferation	[33]
H3K56	Hyperacetylation	P300/CPB	Hyperacetylation is associated with cancer promotion	[29]
H4K8	Hypoacetylation	P300/CPB	Hypoacetylation is associated with cancer promotion	[29]
H4K16	Hypoacetylation	hMOF/Tip60	Hypoacetylation is associated with cancer promotion	[29]
	Hyperacetylation		hMOF promotes acetylation to enhance oral cancer cell growth	[33]

**Table 4:** Histone deacetylase and expression in OSCC.

Name	Expression	Function	References
HDAC2	High expression	Overexpressed in OSCC, resulting in protein HIF-1 *α*, resulting in increased invasion and migration of HNSCC	[39]
HDAC6	High expression	Send *α*-tubulin deacetylation, thus promoting the process of tumor metastasis development	[40, 41]
HDAC7	High expression	Activate c-myc and promote the proliferation of oral tumor cells	[42]
HDAC8	High expression	Inhibition of caspase activation and autophagy in OSCC	[42]
HDAC9	High expression	Promote tumor growth by targeting proapoptotic genes	[43]
SIRT1, 2, 3, 5, 7	Low expression	Inhibit tumor and prevent DNA damage and oxidative stress	[45, 46]

**Table 5:** Antitumor effects of HDACis in OSCC.

Class	Name	Target	Mechanism	References
Hydroxamic acids	SAHA	HDAC I, HDAC II, HDAC IV	Induces hyperacetylation of H2A and *H*3 and inhibits tumor activity	[57]
			Upregulate E calcineurin and ErbB3, downregulate vimentin, EGFR and ErbB2, and enhance the antitumor effect of defibrotide	[58]
	TSA	HDAC I, HDAC II	Induced apoptosis of tumor cells in combination with PS-341	[59]
			Combine with ATRA to inhibit tumor growth	[60]
	LBH589	HDAC I, HDAC II, HDAC IV	Induce high acetylation of *H*3 and *H*4, inhibit tumor growth and induce apoptosis, and lead to *G*1 phase block	[61]
			Upregulate the expression of p21 and induce *G*2/*M* arrest and cancer cell apoptosis	[62]

Cyclopeptides	Apicidin	HDAC1, HDAC3	Competitive combination of HDAC and antitumor proliferation	[63]
			Increase the level of LC3-II and increase the apoptosis and autophagy of tumor cells	[64]
	FK228	HDAC1, HDAC2	Reduce Ki67 staining and inhibit tumor growth	[65]
			Induce high expression of hTERT and inhibit tumor growth	[66]
	Trapoxin	HDAC8	Inhibit tumor cell proliferation as a potential anticancer compound	[67]

Short-chain fatty acids	VPA	HDAC I, HDAC II	Promote *H*3, *H*4 acetylation and inhibit oncogene expression	[68]
			Increase *G*1 arrest and apoptosis of tumor cells	[69]
	PBA	HDAC I, HDAC II	Promote tumor cell apoptosis and inhibit EMT transformation	[70]
			Reduce TNF-*α* level and promote DNA repair	[71]
	NaBu	HDAC I, HDAC IIa	Induce cell cycle arrest, related to the increased expression of kip1	[72]

Benzamides	MS-275	HDAC I	Make tumor cells stagnate in *G*0/*G*1 phase, enhance *H*3 and *H*4 acetylation, and promote apoptosis	[73]
			Increase cisplatin cytotoxicity	[74]
			Inhibit tumor migration and invasion by activating mir-107 and miR-138	[75]
	MGCD0103	HDAC I, HDAC IV	Has better radiotherapy sensitization effect	[76]

## Data Availability

The data used to support the findings of this study are available from the corresponding authors upon request.
